# Efficient synthesis of pillar[5]quinone for rapid identification of aromatic nitro explosives

**DOI:** 10.1039/d6ra06389f

**Published:** 2026-10-12

**Authors:** Yue-Yi Zhang, Lu Wang, Jiabin Wu, Si-Tong Fan, Xin-Yu Che, Yu-Han Wang, Zhi-Yuan Zhang, Yao-Hua Liu, Xing Zhou, Yue Liu, Chunju Li

**Affiliations:** a Academy of Interdisciplinary Studies on Intelligent Molecules, Tianjin Key Laboratory of Structure and Performance for Functional Molecules, College of Chemistry, Tianjin Normal University Tianjin 300387 P. R. China jbwu@tjnu.edu.cn zzy@tjnu.edu.cn hxxyly@tjnu.edu.cn cjli@shu.edu.cn; b College of Aerospace Science and Engineering, National University of Defense Technology Changsha 410073 P. R. China liuyaohua@mail.nankai.edu.cn xingzhou_nudt@nudt.edu.cn

## Abstract

An efficient synthesis of pillar[5]quinone (Q5) is reported for the rapid identification of aromatic nitro explosives. A record-high synthetic yield of 96% on a gram scale for Q5 can be achieved *via* a two-step reaction process involving the demethylation of pillar[5]arene to hydroxypillar[5]arene, followed by oxidation with Oxone to form Q5. In addition, by modifying amino silicon spheres with Q5 to prepare a stationary phase material (Q5-Sil) for high-performance liquid chromatography (HPLC), efficient separation of eight typical aromatic nitro-explosives was achieved using a single column. The underlying mechanism of the separation of aromatic nitro-explosives in the Q5-Sil-packed column resulted from dipole–dipole interactions, hydrophobic effects, and steric factors between Q5 and analytes. This work achieved the quantitative synthesis of Q5, established a novel method of macrocyclic modification of chromatographic column stationary phase materials, and provided a mixed mechanism for the HPLC-based separation of aromatic nitro-explosives, expanding the interdisciplinary applications of macrocyclic supramolecular chemistry in the field of analytical separation science.

## Abstract 2

An optimized synthetic scheme for pillar[5]quinone (Q5) was developed, affording a 96% yield. Q5 was further applied as the stationary-phase material for an HPLC column, enabling the separation of eight aromatic nitro-explosives in one column.

## Graphical abstract

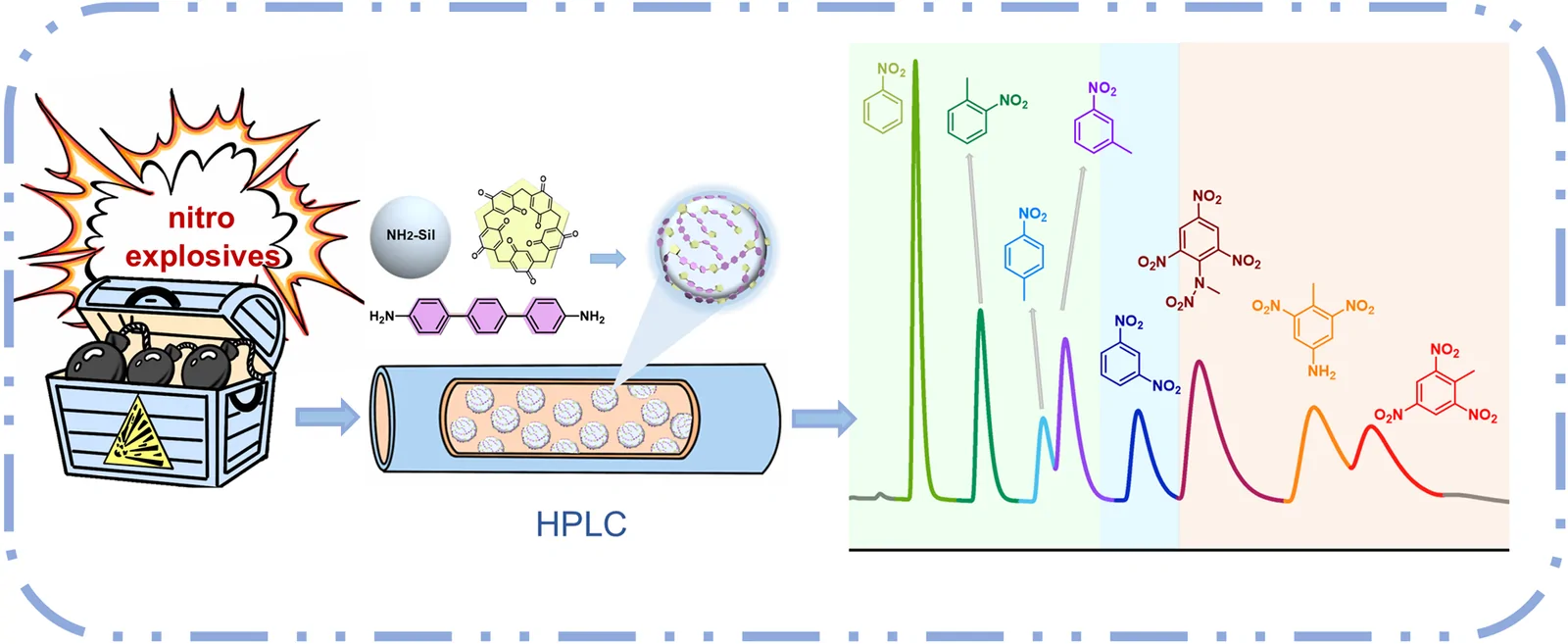

## Introduction

With the continuous exacerbation of international terrorism, numerous explosives with complex compositions have been used.^1,2^ Among them, aromatic nitro compounds are one of the mainstream types of explosives, and they pose a serious threat to social stability.^3,4^ Therefore, it is crucial to develop methods with high efficiency and specificity for the detection and separation of aromatic nitro-explosives.^5,6^ Current detection techniques for aromatic nitro-explosives mainly include fluorescence spectroscopy (optical sensors),^7–9^ gas chromatography coupled with mass spectrometry (GC-MS),^10,11^ and high-performance liquid chromatography coupled with a photodiode array detector (HPLC-PDA).^12–14^ Some features of aromatic nitro-explosives, such as their thermolability and high boiling points, are incompatible with gas chromatography, making HPLC-based methods more suitable for their detection due to their high resolution, sensitivity, and repeatability. However, there is still a paucity of research on the use of HPLC to analyze aromatic nitro-explosives because of their complex composition.^15^ The National Environmental Protection Agency of the United States uses two-dimensional HPLC to separate aromatic nitro-explosives. However, using two in-series columns can lead to high costs and cumbersome processes. Thus, achieving the efficient separation of various aromatic nitro-explosives with a single column is quite attractive. The separation capacity of HPLC relies on efficient columns, so it is crucial to design a novel stationary phase specifically for aromatic nitro-explosive separation.

Macrocyclic compounds, including crown ethers,^17,18^ cyclodextrins,^19–22^ calix[*n*]arenes,^23–25^ cucurbit[*n*]urils,^26–28^ pillar[*n*]arenes,^29–32^*etc.*,^33–41^ are the fundamentals of modern supramolecular chemistry.^16^ The preorganized cavities and multiple recognition sites of macrocycles provide excellent host–guest binding properties, making them superior tools in molecular recognition and adsorption.^42–48^ Accordingly, macrocycle-modified silica stationary phases have been developed to achieve chromatographic selectivity beyond that of conventional alkyl-bonded silica.^49–53^ In particular, stationary phases based on cyclodextrins, calixarenes, and pillar[*n*]arenes have been successfully applied to the separation of chiral compounds, polycyclic aromatic hydrocarbons, phenols, and aromatic positional isomers. Nevertheless, the chromatographic separation of aromatic nitro explosives remains challenging because these analytes often possess closely related structures and physicochemical properties. Their retention may be affected by the number and position of nitro groups, molecular hydrophobicity, substituent effects, and molecular shape. Q5 has a rigid macrocyclic framework containing ten quinone carbonyl groups. Its carbonyl-rich surface and intrinsic cavity may provide multiple recognition sites for aromatic nitro compounds. Moreover, its quinone units can react with amino-containing linkers, facilitating immobilization on amino-functionalized silica.^54^ Q5 has also been incorporated into functional materials for molecular detection, adsorption, separation,^55,56^ and radioactive organic iodide capture, demonstrating its potential in guest-responsive materials.

Several methods have been reported for preparing Q5 from pillar[5]arene derivatives using ceric ammonium nitrate, hypervalent iodine reagents, and other oxidation systems.^31,57–60^ These methods differ in oxidant requirements, reaction time, isolated yield, reaction scale, and purification procedure. A recent CAN-mediated direct oxidation, for example, afforded Q5 in 92% yield within 30 min.^59^ Therefore, alternative routes should be evaluated not only in terms of reaction time and yield, but also scalability, reproducibility, and ease of product isolation.

Herein, we report a two-step reaction for the synthesis of Q5 in a record synthetic yield of 96% on a gram scale. Moreover, the Q5 was modified onto NH_2_-Sil as a stationary phase material for HPLC, and eight typical aromatic nitro-explosives selected primarily from EPA Method 8330A could be resolved in a single column packed with this material. Comparative retention experiments were also conducted to examine the combined contributions of dipole–dipole interactions, hydrophobic effects, and steric factors. This work extends Q5-based stationary phases to nitro-explosive separation and provides preliminary insight into the relationship between analyte structure and chromatographic retention. This research provides a new insight into the unique advantages of macrocyclic compounds in analytical separation science.

## Results and discussion

Previous studies have established several direct oxidative methods for the synthesis of Q5 from pillar[5]arene derivatives. These methods differ in their oxidants, reaction times, reaction scales, isolated yields, and purification procedures. In the present work, we employed a stepwise strategy in which pillar[5]arene was first converted into hydroxypillar[5]arene, followed by oxidation to Q5. Separating the demethylation and oxidation processes allowed the Q5-forming oxidation step to be optimized independently.

The crude hydroxypillar[5]arene intermediate was used directly in the subsequent oxidation without chromatographic purification.^61,62^ The oxidation conditions were then optimized with respect to the Oxone loading, iodobenzene loading, acetonitrile/water ratio, and reaction time. Sufficient water was required to dissolve Oxone and facilitate complete oxidation, whereas the presence of iodobenzene substantially improved the conversion. Under the optimized conditions, Q5 was obtained on the gram scale in an isolated yield of 96% and was isolated by filtration and washing. Therefore, the practical features of this method lie in its stepwise controllability, gram-scale applicability, and operationally simple isolation.

These observations motivated us to investigate the oxidation of hydroxypillar[5]arene to Q5 and to examine the effects of oxidants, solvents, and reaction time on the overall yield. As shown in Table 1, several conclusions could be drawn: (1) the presence of iodobenzene was essential. Without an iodine source, the yield decreased significantly (Table 1; entries 2 and 5), which might be attributed to the lack of highly reactive hypervalent iodine compounds. Ochiai *et al.* indicated that iodobenzene could be oxidized to produce labile (diacyloxyiodo)benzenes, which facilitated the acetoxylation of ketones.^63^ (2) The reaction was water-dependent. Lower proportions of water in acetonitrile were unfavourable for this reaction (Table 1; entries 8 and 9), because a small amount of water could not dissolve Oxone sufficiently, resulting in incomplete oxidation of hydroxypillar[5]arene. When the volume ratio of acetonitrile to water was 1 : 1 (v/v), the reaction yield reached 93%. However, further increase in the water content in the solvent did not have a significant influence on the reaction (Table 1; entries 6 and 8). (3) Using a predetermined temperature (25 °C) and reaction time (2 days), the amount of Oxone was varied. We found that 10 equiv. of Oxone resulted in the optimal yield (Table 1; entries 2–4), and a longer reaction time could compensate for a decreased amount of Oxone, leading to nearly complete conversion (Table 1; entries 4 and 11). Finally, we determined the optimal reaction conditions for the preparation of Q5. Oxone (10 eq.) and iodobenzene (2.5 eq.) were used as oxidants, and a 1 : 1 (v/v) mixture of acetonitrile and water served as the solvent. The reaction temperature was maintained at 25 °C, and the reaction time was set to 2 days.

**Table 1:** Screening of reaction conditions for Q5

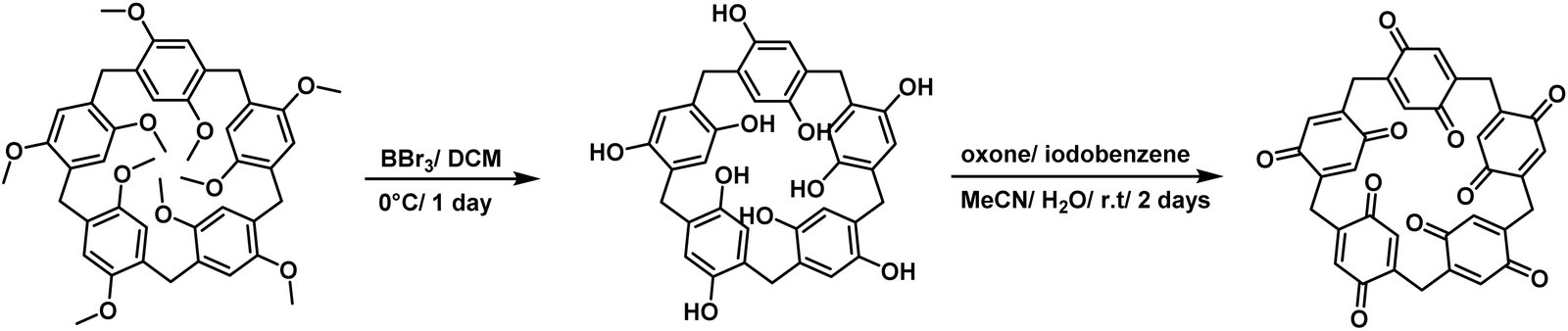						
Entry	Synthesis method	Time (day)	Solvent (V : V)	Oxone (eq.)	Iodobenzene (eq.)	Yield (%)
1	One-step oxidation^a^	3.0	CH_3_CN : H_2_O 1 : 1	10	1.5	33
2	Stepwise oxidation	3.0	CH_3_CN : H_2_O 1 : 1	10	1.5	93
3	Stepwise oxidation	3.0	CH_3_CN : H_2_O 1 : 1	20	1.5	92
4	Stepwise oxidation	3.0	CH_3_CN : H_2_O 1 : 1	5.0	1.5	85
5	Stepwise oxidation	3.0	CH_3_CN : H_2_O 1 : 1	10	0.0	41
6	Stepwise oxidation	3.0	CH_3_CN : H_2_O 1 : 1	10	2.5	93
7	Stepwise oxidation	3.0	CH_3_CN : H_2_O 1 : 1	10	3.5	93
8	Stepwise oxidation	3.0	CH_3_CN : H_2_O 1 : 5	10	2.5	89
9	Stepwise oxidation	3.0	CH_3_CN : H_2_O 5 : 1	10	2.5	40
10	Stepwise oxidation	0.5	CH_3_CN : H_2_O 1 : 1	10	2.5	74
11	Stepwise oxidation	1.0	CH_3_CN : H_2_O 1 : 1	10	2.5	92
12	Stepwise oxidation	2.0	CH_3_CN : H_2_O 1 : 1	10	2.5	96

a 33% yield with the oxidant Oxone;^57^ 78% yield with the oxidant CAN.^58^

Next, we modified the commercially available stationary phase with Q5 *via* a one-step reaction. Briefly, NH_2_-Sil, benzidine, and Q5 were mixed in ethanol and refluxed for 3 days to obtain a modified stationary phase (Q5-Sil),^53^ which was then washed with ethanol (Fig. 1a). Thermogravimetric analysis showed that the weight loss of Q5-Sil at 100 °C was only 2.5%, indicating that the modified stationary phase could remain stable under HPLC conditions. Moreover, the weight loss of Q5-Sil was 17.5% at 600 °C, at which covalent bonds between the original stationary phase and modified Q5 could be completely cleaved, and this result revealed the loading capacity of NH_2_-Sil to Q5 (Fig. 1b). Additionally, Fourier Transform Infrared (FT-IR) spectroscopy was employed to characterize the Q5-Sil microspheres. The FT-IR spectrum of Q5 displayed a characteristic peak at 1660 cm^−1^, representing its carbonyl groups (Fig. S3). The major characteristic peak in the FT-IR spectra of both NH_2_-Sil and Q5-Sil at 1060 cm^−1^ represented the Si–O bond from silicon spheres, while the additional characteristic peaks at 1600 cm^−1^ (–C=O) and 1440 cm^−1^ (–C–N–) in the Q5-Sil spectrum evidenced the covalent interaction between the silicon spheres of NH_2_-Sil and Q5, demonstrating that Q5 was modified on the surface of the NH_2_-Sil (Fig. 1c).

**Fig. 1:**
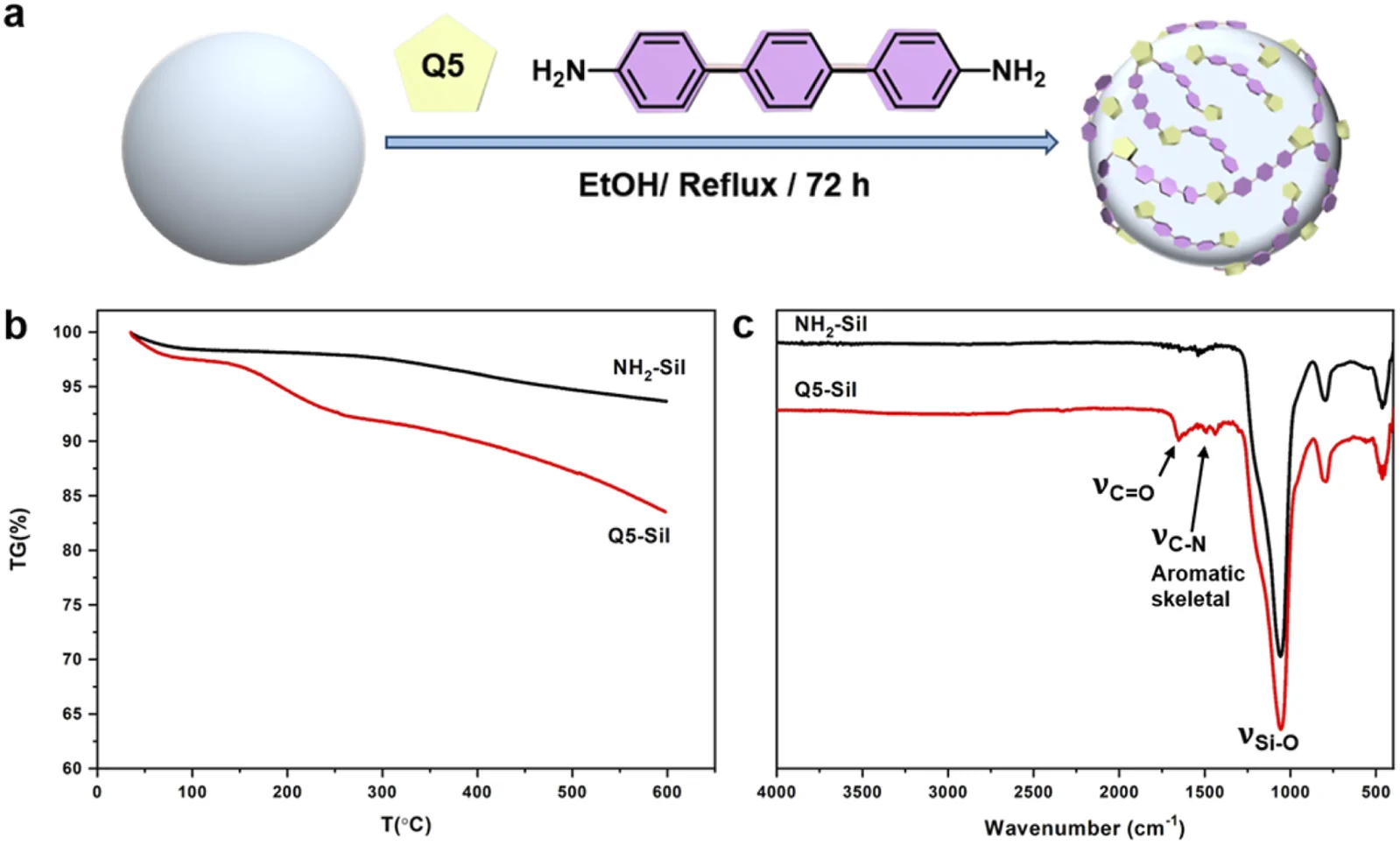
(a) Reaction scheme of Q5-Sil. (b) Thermogravimetric analysis of NH_2_-Sil and Q5-Sil. (c) FT-IR spectra of NH_2_-Sil and Q5-Sil.

After the characterization of Q5-Sil, we tested its separation capacity for benzene rings with different substituents on HPLC. By controlling the proportion of methanol in the mobile phase, the interaction between Q5 and these analytes could be regulated to achieve efficient separation. First, we observed that the Q5-Sil-packed column had the best resolutions for nitrobenzene derivatives. *o*-, *m*-, and *p*-Dinitrobenzenes, *o*-, *m*-, and *p*-nitrobenzonitriles, and *o*-, *m*-, and *p*-nitrophenols could achieve baseline separation, unlike nitrobenzaldehydes (Fig. 2a–d). Additionally, the Q5-Sil-packed column also had great separation performance toward *o*-, *m*-, and *p*-benzenediols and *o*-, *m*-, and *p*-dicyanobenzenes (Fig. S4 and S5). However, none of the tested aniline derivatives (*o*-, *m*-, and *p*-aminophenol, *o*-, *m*-, and *p*-aminobenzoate, and *o*-, *m*-, and *p*-fluoroaniline) could be resolved in the modified column (Fig. S6). The common amino group of these analytes may form strong but poorly differentiated hydrogen-bonding and polar interactions with the quinone carbonyl groups of Q5. Such interactions may dominate their retention and mask the comparatively subtle differences associated with substituent position, molecular shape, and hydrophobicity. Moreover, solvation and possible partial protonation of the aniline derivatives in aqueous methanol may interfere with well-defined cavity-mediated recognition. Therefore, the positional isomers do not establish sufficiently distinct multipoint interaction geometries with Q5-Sil to achieve baseline separation. From the HPLC traces, the retention factors, selectivity factors, and resolutions of each group of substituted benzenes were calculated and plotted on radar charts (Fig. 2e, S4e, S5e, S6e and Tables S2–S5). In Fig. 2e, *r* and *R* represent the relative retention factors and resolutions among *o*-, *m*-, and *p*-disubstituted benzenes, respectively. The areas enclosed by these four dimensions represent the overall separation performance among the analytes. Compared with the other three groups of substituted benzenes (anilines, cyanobenzenes, and benzenediols), the nitrobenzene derivatives had the best separation efficiencies (Fig. 2e, S4e, S5e and S6e). These results revealed that Q5-Sil had the potential to be an effective stationary phase for HPLC separation of nitro explosives.

**Fig. 2:**
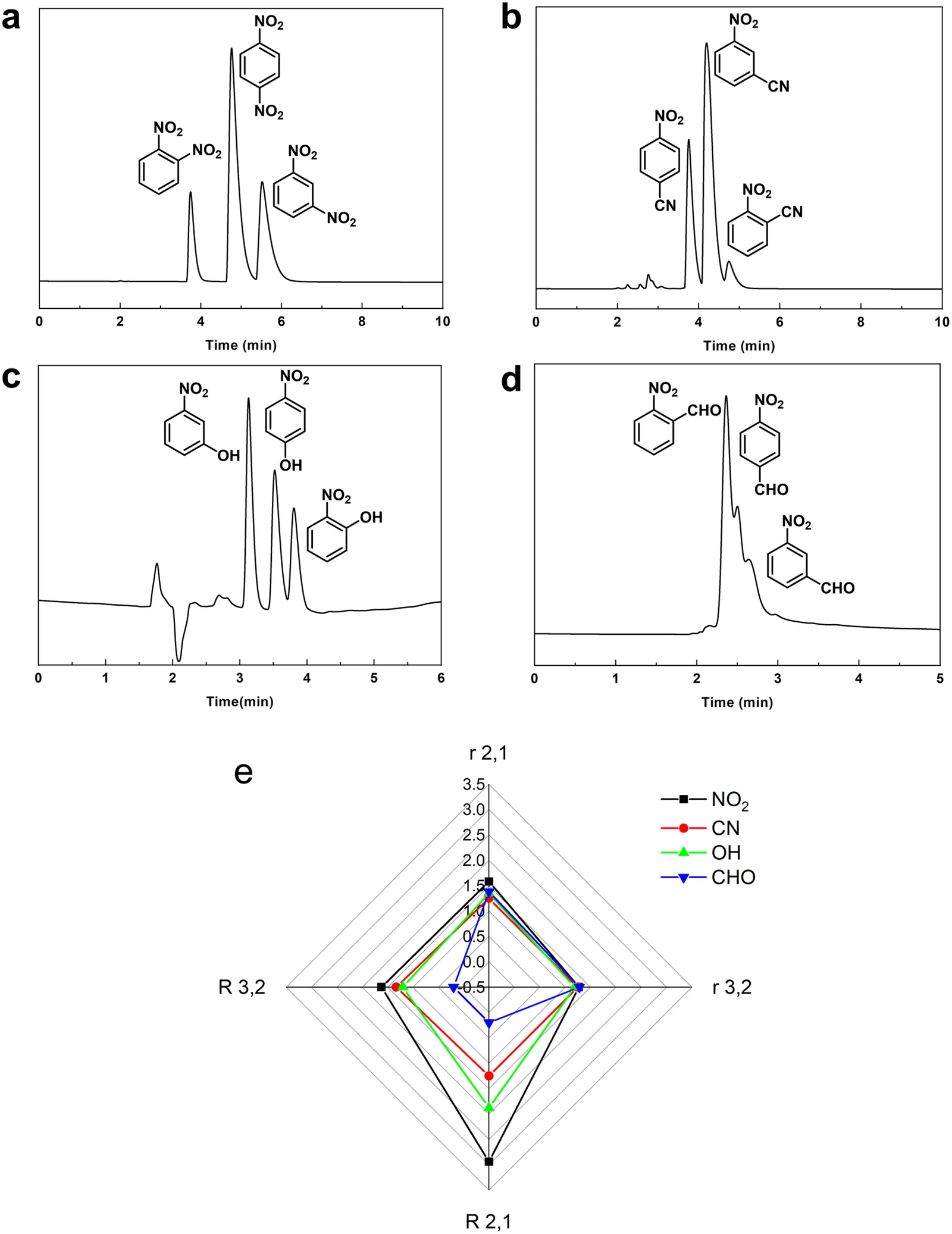
Separation of (a) dinitrobenzenes, (b) nitrobenzonitriles, (c) nitrophenols, and (d) nitrobenzaldehydes on the Q5-Sil packed column. The corresponding analytes are identified by their chemical structures above the peaks. (e) Effect of nitro-group-functionalized positional isomers on the Q5-Sil packed column (*r* represents relative retention value, *R* represents resolution).

Next, we employed the Q5-Sil-packed column to separate eight typical nitrobenzene derivatives. The mobile-phase composition was first optimized by varying the methanol content from 70% to 50% while maintaining the AcOH content at 2.5% (Fig. S7b). Decreasing the methanol content generally increased analyte retention and improved the separation of the eight compounds. At higher methanol contents, several analytes eluted rapidly and exhibited insufficient resolution. Based on the overall separation performance, MeOH/H_2_O/AcOH = 50 : 47.5 : 2.5 (v/v/v) was selected for the subsequent chromatographic experiments. The eight analytes included nitrobenzene, 2-nitrotoluene, 4-nitrotoluene, 3-nitrotoluene, *m*-dinitrobenzene, 2,4,6-trinitrobenzene, 2-amino-4,6-dinitrotoluene, and trinitrotoluene. This column had better retention and separation performance for these nitrobenzene derivatives than the unmodified NH_2_-Sil column (Fig. 3a, S7a and Table S6), which implied that the Q5 polymer might play a crucial role in the separation of nitro explosives. Additionally, the retention times of the aromatic nitro-explosives were related to the number of nitro substituents. The retention times of the eight analytes generally increased with increasing structural complexity and number of nitro groups. First, the elution order generally followed the sequence mononitro compounds < dinitro compounds < trinitro compounds. An increase in the number of nitro groups provides additional polar interaction sites and changes the molecular dipole distribution, potentially strengthening the overall dipole–dipole interactions with the quinone carbonyl groups of Q5, eventually causing a longer retention time. Second, the three nitrotoluene isomers exhibited longer retention times than nitrobenzene, suggesting that the hydrophobic methyl group enhances the affinity of the analytes for the Q5-Sil stationary phase. Third, the different retention times of 2-, 3-, and 4-nitrotoluene demonstrate that the position of the substituent and the resulting steric environment influence the accessibility and spatial orientation of the analytes at the stationary-phase surface. Similarly, the distinct retention behaviors of 1,3-dinitrobenzene, tetryl, 2-amino-4,6-dinitrotoluene, and trinitrotoluene may reflect differences in their nitro-group distribution, hydrophobicity, and molecular geometry. Therefore, the separation of these analytes on Q5-Sil is likely governed by the combined contributions of dipole–dipole interactions, hydrophobic effects, and steric factors, rather than by any single interaction.

**Fig. 3:**
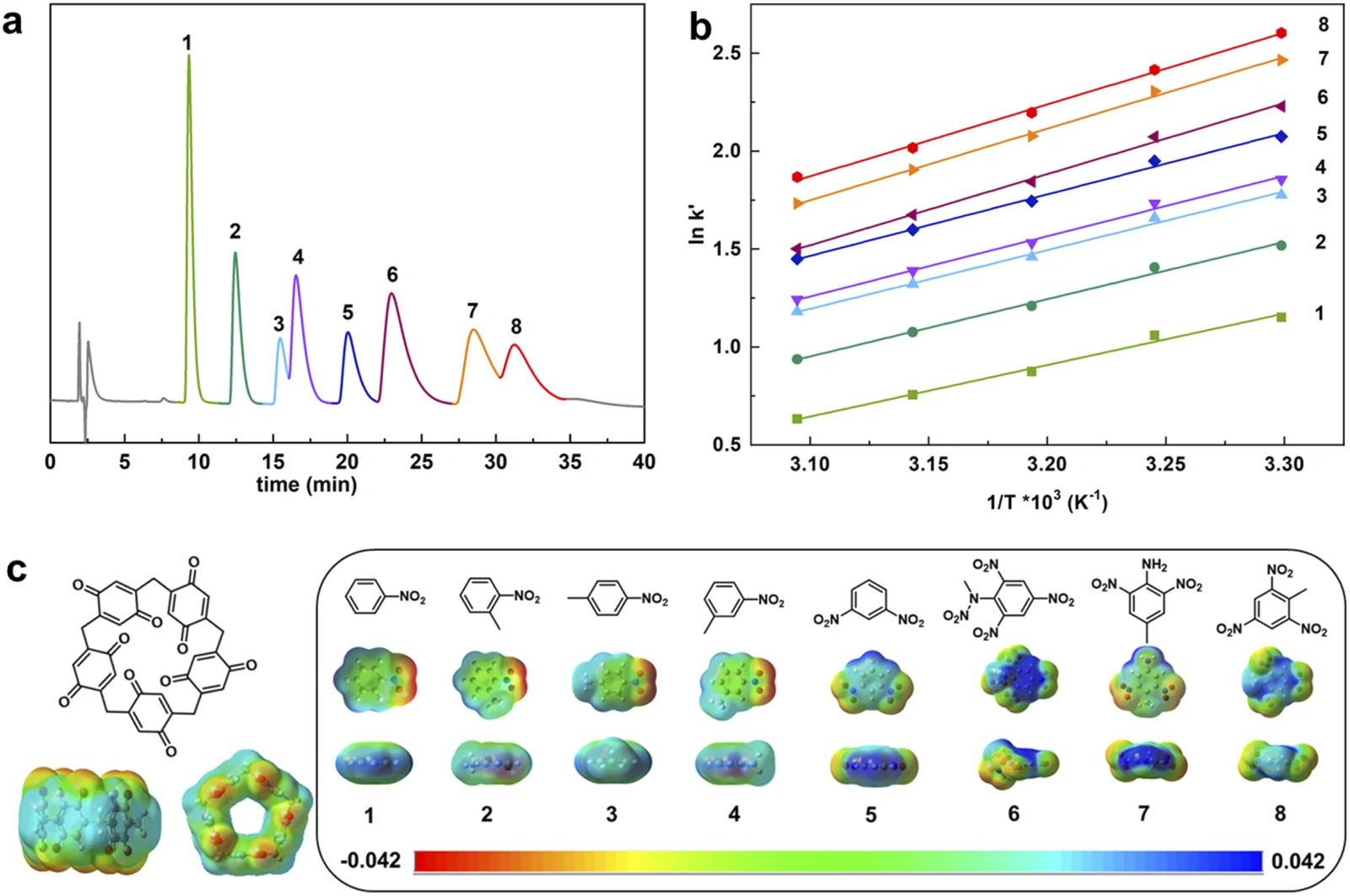
(a) Chromatogram of a Q5-Sil packed column (2.1 mm × 150 mm, 5 µm) for the LC separation of explosives under MeOH/H_2_O/AcOH = 50/47.5/2.5 (v/v/v) at a flow rate of 0.2 mL min^−1^. (b) van't Hoff plots for explosives on the Q5-Sil packed column. (c) Electrostatic simulation of the surface of Q5 and the aromatic nitro-explosives. (Peak 1: nitrobenzene, Peak 2: 2-nitrotoluene, Peak 3: 4-nitrotoluene, Peak 4: 3-nitrotoluene, Peak 5: *m*-dinitrobenzene, Peak 6: 2,4,6-trinitrophenylmethylnitramine, Peak 7: 2-amino-4,6-dinitrotoluene, Peak 8: trinitrotoluene).

Moreover, the adsorption enthalpies between Q5-Sil and the aromatic nitro-explosives were calculated to elucidate the underlying adsorption mechanism. The aforementioned eight nitro explosive components were adsorbed and desorbed on a Q5-Sil-packed column, and retention data were collected at 30–50 °C using PDA detection, from which the thermodynamic parameters were derived using the van't Hoff relationship. The adsorption enthalpy changes (Δ*H*) and entropy changes (Δ*S*) of each analyte on Q5-Sil were obtained from the van't Hoff equation (eqn (1), where *k*′ is the retention factor, *T* is the absolute temperature, *R* is the universal gas constant, and *Φ* is the ratio of the volume of the stationary phase to that of the mobile phase). Additionally, the Gibbs free energy changes (Δ*G*) from the mobile phase to the stationary phase of the adsorbent were calculated using eqn (2):1
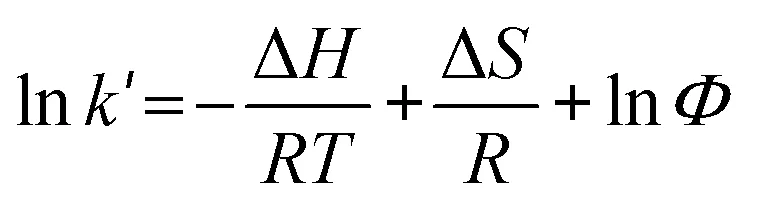

2 Δ*G* = Δ*H* − *T*Δ*S*

As shown in Fig. 3b and Table S7, linear relationships between ln *k*′ and 1/*T* were obtained for all eight analytes over the investigated temperature range, with *R*^2^ values of 0.988–0.997. The positive slopes of the van't Hoff plots indicate that retention decreased with increasing temperature. Correspondingly, the apparent Δ*H* values were negative, ranging from −21.8 to −30.5 kJ mol^−1^, confirming that the transfer of the analyte from the mobile phase to Q5-Sil was exothermic and enthalpically favorable. The increasingly negative apparent Δ*H* values observed for the more strongly retained analytes suggest greater overall energetic contributions to retention. All the apparent Δ*S* values were also negative, which may reflect restricted molecular freedom following transfer to the stationary phase, together with changes in analyte solvation and desolvation. The negative apparent Δ*G* values indicate that this transfer was thermodynamically favorable under the investigated conditions. Overall, the apparent thermodynamic parameters provide an energetic description of analyte retention on Q5-Sil, which involves the combined contributions of analyte-stationary-phase interactions and mobile-phase effects. In addition, no obvious color change was observed after grinding Q5 and 4-nitrotoluene in a 1 : 1 molar ratio in the presence of methanol (Fig. S7d), and no new absorption band appeared in the UV-vis spectrum of their mixture (Fig. S7d). These observations indicate that no detectable ground-state charge-transfer complex was formed under the examined conditions but do not exclude other non-covalent interactions. The trends in the apparent Δ*G* values were qualitatively consistent with the observed elution order.

## Conclusion

In conclusion, the high-yield synthesis (96%) of Q5 on a gram-scale was achieved *via* a two-step reaction from pillar[5]arene. The first step, *i.e.*, the demethylation of pillar[5]arene, could boost the subsequent oxidation reaction mediated by Oxone and iodobenzene. The appropriate ratio of acetonitrile to water is also important for achieving a high reaction yield. This method laid the foundation for the preparation of low-cost column stationary phase materials. Subsequently, Q5-modified HPLC stationary phase materials were prepared, and the single-column separation of a variety of aromatic nitro-explosives was accomplished based on a novel mixed mechanism involving dipole–dipole interactions, hydrophobic effects, and steric factors between Q5 and explosives. These results provide new insight into the design of high-efficiency liquid chromatographic columns and show that the Q5-Sil column is expected to be able to predict the danger level of unknown explosives by measuring their retention times and the elution sequences.

## Author contributions

Yue-Yi Zhang: data curation, formal analysis, investigation, software, validation, visualization, writing – original draft. Lu Wang: formal analysis, investigation, validation, visualization. Jiabin Wu: conceptualization, methodology, funding acquisition, project administration, resources, supervision, writing – review and editing. Si-Tong Fan: formal analysis, investigation. Xin-Yu Che: formal analysis, investigation. Yu-Han Wang: investigation. Zhi-Yuan Zhang: conceptualization, funding acquisition, methodology, project administration, resources, supervision. Yao-Hua Liu: resources, supervision. Xing Zhou: resources, supervision. Yue Liu: conceptualization, formal analysis, investigation, supervision. Chunju Li: conceptualization, funding acquisition, resources, supervision.

## Conflicts of interest

There are no conflicts to declare.

## Supplementary Material

RA-016-D6RA06389F-s001

## Data Availability

The data supporting this article have been included as part of the supplementary information (SI). Supplementary information: the electrostatic simulation of the surfaces of nitro-explosives and Q5, the synthesis and characterization of Q5 and Q5-Sil, and the high-performance liquid chromatography separation of each analyte on the Q5-Sil packed column. See DOI: https://doi.org/10.1039/d6ra06389f.
