# Supplementary material for: Efficient synthesis of pillar[5]quinone for rapid identification of aromatic nitro explosives
Source: RSC Adv. 2026 Oct 12;16(47):62226–32. doi: 10.1039/d6ra06389f (PMC13656025; doi:10.1039/d6ra06389f)
Supplement: Supplementary file 1 [file RA-016-D6RA06389F-s001.pdf]

Supplementary Information (SI) for RSC Advances.
This journal is © The Royal Society of Chemistry

Supporting Information

Efficient Synthesis of Pillar[5]quinone for Rapid Identification of Aromatic Nitro Explosives

Yue-Yi Zhang,[a†] Lu Wang,[a†] Jiabin Wu,[,a] Si-Tong Fan,[a] Xin-Yu Che,[a] Yu-Han Wang,[a] Zhi-Yuan Zhang,[a] Yao-Hua Liu,[*,b] Xing Zhou,[*,b] Yue Liu,[*,a] and Chunju Li[a]*

a. Academy of Interdisciplinary Studies on Intelligent Molecules, Tianjin Key Laboratory of Structure and Performance for Functional Molecules, College of Chemistry, Tianjin Normal University, Tianjin 300387 (P. R. China).

b. College of Aerospace Science and Engineering, National University of Defense Technology, Changsha 410073 (P. R. China)

[†]These authors contributed equally to this work.

Corresponding Author

*E-mail: zzy@tjnu.edu.cn; hxxyly@tjnu.edu.cn; cjli@shu.edu.cn

Table of Contents

Section I. Materials/Methods/Instrumentation..3

Section II. Electrostatic simulation of the surface of nitro-explosives and Q5 4

Section III. Synthetic Protocols of Q5 and Q5-Sil..4-7

Section IV. High-performance liquid chromatography separation...................................8-14

Section I. Materials/Methods/Instrumentation

Unless otherwise noted, materials were obtained from commercial suppliers and were used without further purification. Column chromatography was performed on silica chromatography 300-400 mesh. ^{1}H NMR spectra were recorded on Bruker Avance III 400 MHz in $CDCl_3$. High-performance liquid chromatography separation experiments were performed on Waters ACQUITY UPLC H-Class Ultra-high performance Liquid chromatograph (Waters Technologies (Shanghai) Company Limited) equipped with Waters ACQUITY UPLC photodiode array detector. Empower 3 chromatographic workstation was used for signal acquisition and data processing. The thermogravimetric determination is performed on the NETZSCH synchronous thermal analyzer (NETZSCH STA 2500 Regulus, NETZSCH GMBH, Germany). The thermogravimetric stability of Q5-Sil was investigated by recording the weight change of Q5-Sil from 40 °C to 600 °C at a heating rate of 10 °C/min under N_2 protection. Fourier Transform infrared (FT-IR) spectroscopy (4000-400 cm^{-1}) was measured on the Nicolet 6700 spectrometer (Thermo, USA).

Pillar[5]arene was synthesized according the reported method [1]

Section II. Electrostatic simulation of the surface of nitro-explosives and Q5.

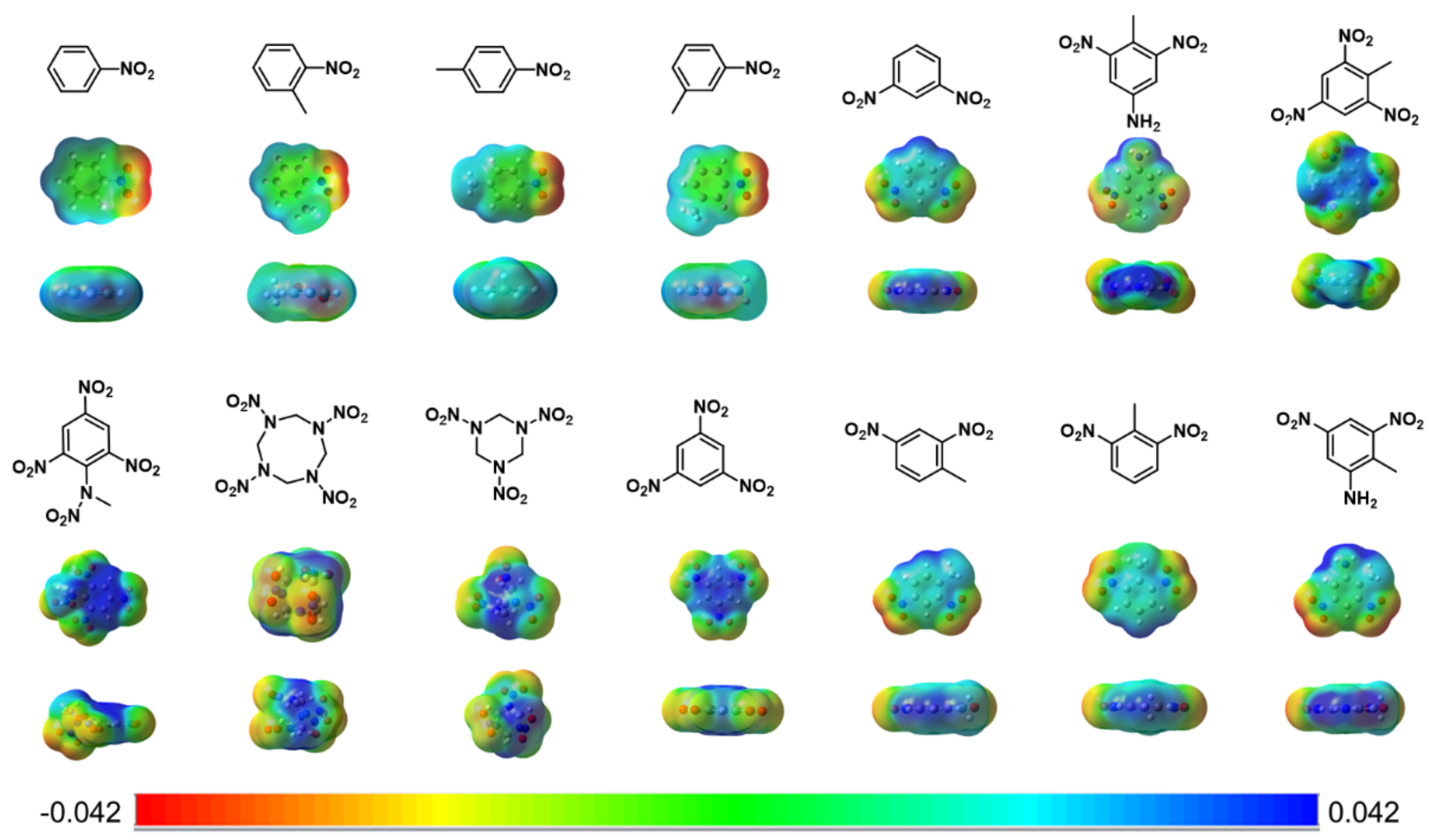

Figure S1. Electrostatic simulation of the surface of nitro-explosives.

Section III. Synthetic Protocols of Q5 and Q5-Sil.

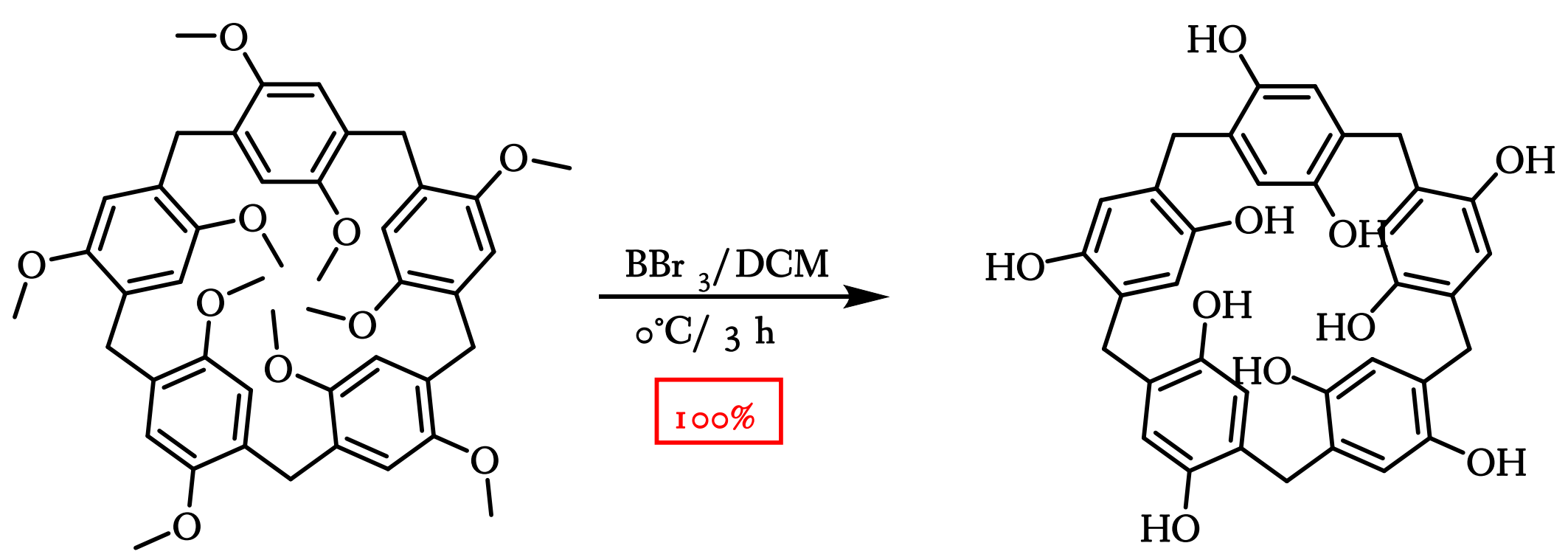

Scheme S1. Synthesis of hydroxypillar[5]arene.

1,4-dimethoxypillar[5]arene (2.0 g, 2.6 mmol) was added into a single-mouth round-bottom flask, methylene chloride (70 mL) was added as the solvent, and the mixture was stirred in an ice water bath. Boron tribromide (6.7 mL, 26 mmol) was added under nitrogen protection and stirred at room temperature for 3 h (the solution was brown-yellow).[2] After the reaction, 200 mL of ice-water mixture was poured into a 500 mL beaker, and the reaction liquid was

stirred and poured into the ice-water mixture. The solution turned brown, a large amount of brown solid was precipitated, and the brown solid was obtained by pumping and filtration, and the water was completely removed by drying at 45 ℃ in a vacuum drying oven. The crude product obtained is no longer post-processed, and is directly put into the next reaction.

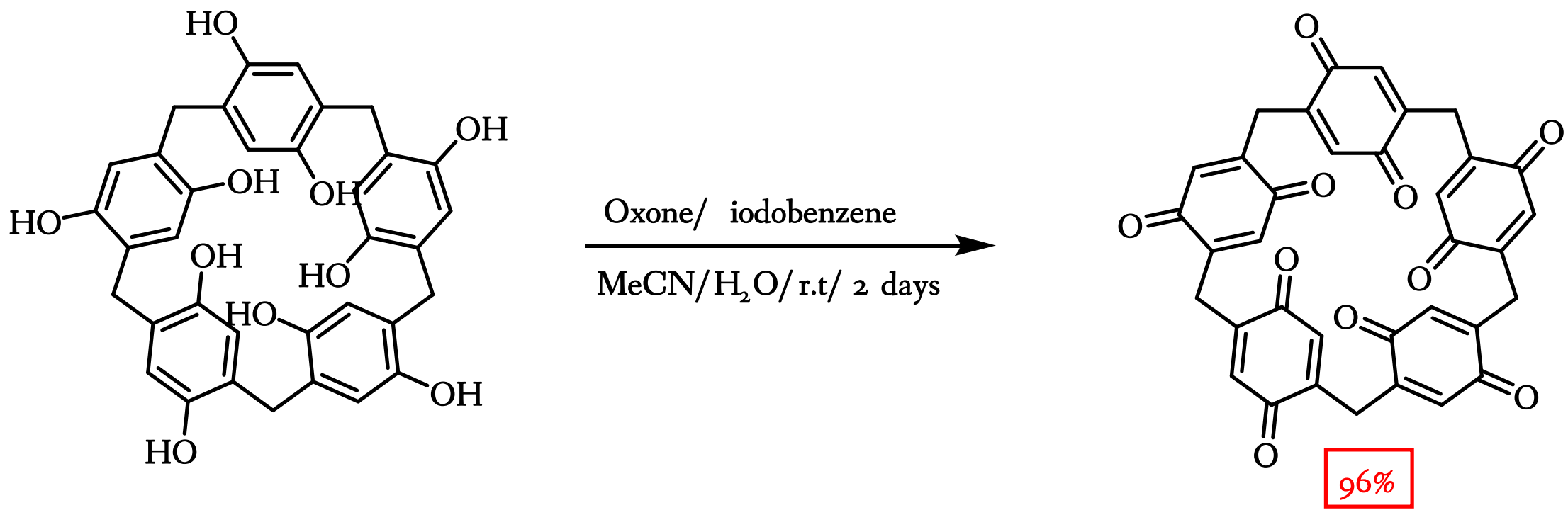

Scheme S2. Synthesis of Q5.

Hydroxypillar[5]arene (2.0 g, 3.2 mmol), potassium monosulfate peroxide and sulfuric acid (20.0 g, 32 mmol) were added successively into the round-bottom flask, then acetonitrile-water (30 mL +30 mL) mixed solvent was added, stirred at room temperature, and iodobenzene (1.7 g, 8.3 mmol) was added. After 2 days of intense stirring reaction at room temperature, treatment (solution from white to golden brown). After the reaction was over, water was added and stirred for 2 hours, then pumped and filtered, and the solid was washed with methanol and a small amount of methylene chloride respectively, and finally yellow solid was obtained, which was dried in a vacuum drying oven at 55 ℃ to completely remove the residual solvent. The Q5 was obtained (1.84 g, 96%). Characterization data: 1H NMR (400 MHz, $CDCl_3$) δ (ppm): 6.81 (s, 10H), 3.59 (s, 10H).

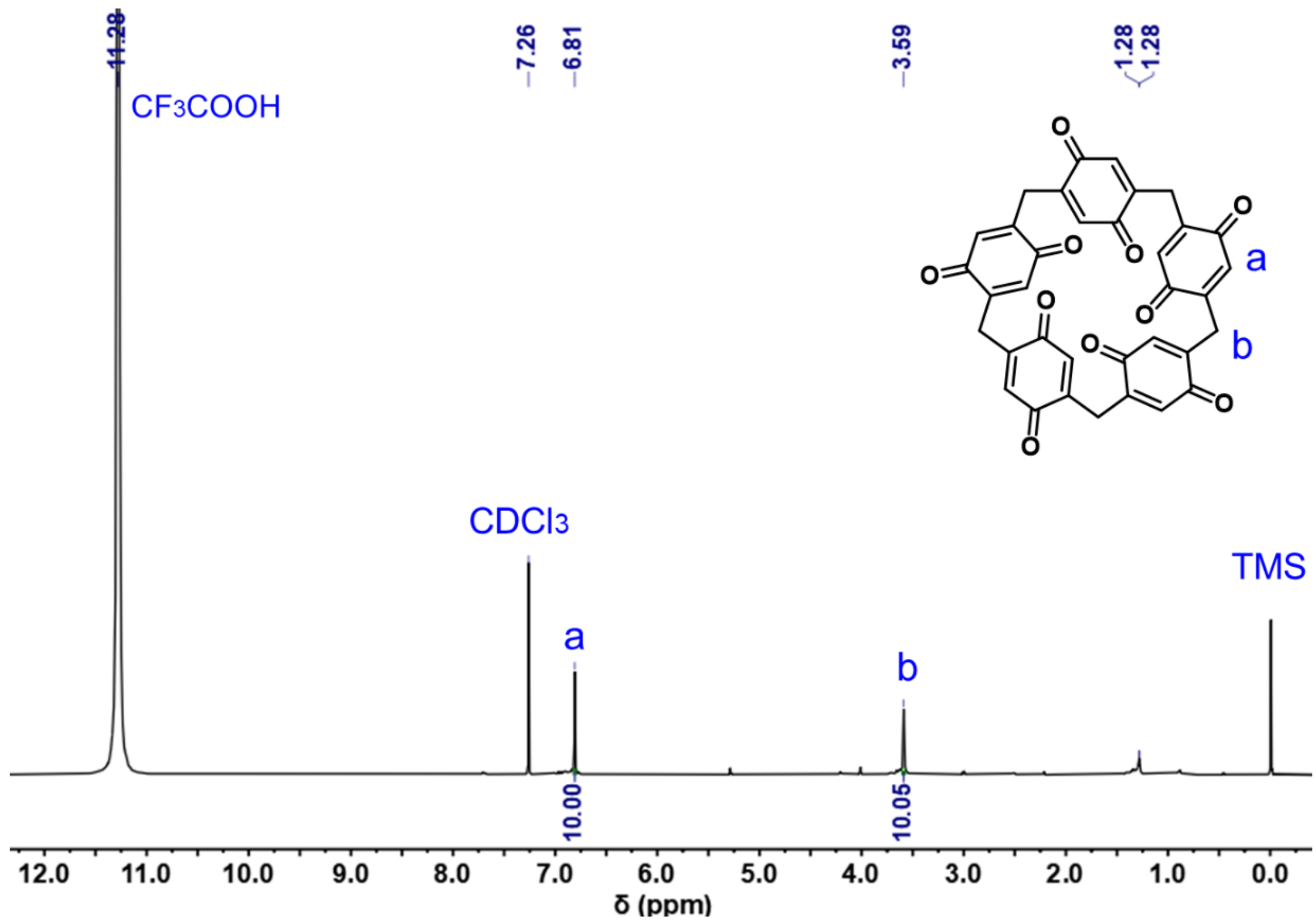

Figure S2. ^{1}H NMR spectrum (400 MHz, $CDCl_3$, 298 K) of Q5.

Q5-Sil stationary phase preparation: 1.0 g of NH_2-Sil, 0.1 g of 4,4'-diaminotriphenylene and 0.5 g of Q5 were mixed with ethanol and refluxed for 72 h. The crude product of Q5-Sil was prepared. The crude product of Q5-Sil was washed with ethanol for several times and filtered to obtain pure Q5-Sil. The product was dried in a vacuum drying oven at 60 °C for 24 h.

Weigh 0.6 g of Q5-Sil into a 100 mL beaker, add 50 mL of methanol, and disperse with ultrasound for 5min. Pour the suspension into a homogenizing tank with a stainless steel column (150 mm×2.1 mm i.d.) at the lower end, mount the homogenizing tank on a column loading machine, load the column at 6000 psi, and then remove the pressure and detach the column. After the column was unloaded, the column was removed and the cap was screwed on, resulting in a Q5-Sil packed column. The column was connected to a Waters ACQUITY UPLC H-Class chromatograph and equilibrated with 0.2 mL/min methanol for 2 h for

chromatographic separation. The mobile phase was methanol: water: glacial acetic acid (50: 47.5: 2.5) at a flow rate of 0.2 mL/min with an injection volume of 2 μL. The detection wavelength was 254 nm, and the temperature of the column oven was set at 30 ℃.

Table S1. Comparison of representative synthetic methods for pillar[5]quinone (Q5).

Starting material	Oxidation system	Scale	yield	Reference
P5	Ceric ammonium nitrate (CAN)	/	62%	*Angew. Chem. Int. Ed.* 2009, **48**, 9721–9723.
P5	Oxone(12 eq.) /iodobenzene (2eq.)	25.0 g starting material	33%	*Synthesis.* 2013, **45**, 896–898
P5	CAN (11 equiv. for complete oxidation)	1.0 g starting material	78%	*New J. Chem.*, 2015, **39**, 9215–922
P5	Oxone/iodobenzene	2.0 g starting material	48%	*ACS Appl. Mater. Interfaces*, 2022, **14**,810–6817
P5	CAN (15–18 eq.)	/	92%	*Tetrahedron Lett.*, 2025,**169**, 155739
P5 via P5-OH	Step 1: BBr₃; Step 2: Oxone (10 equiv.)/iodobenzene (2.5 equiv.)	2.0 g	96%	This work.

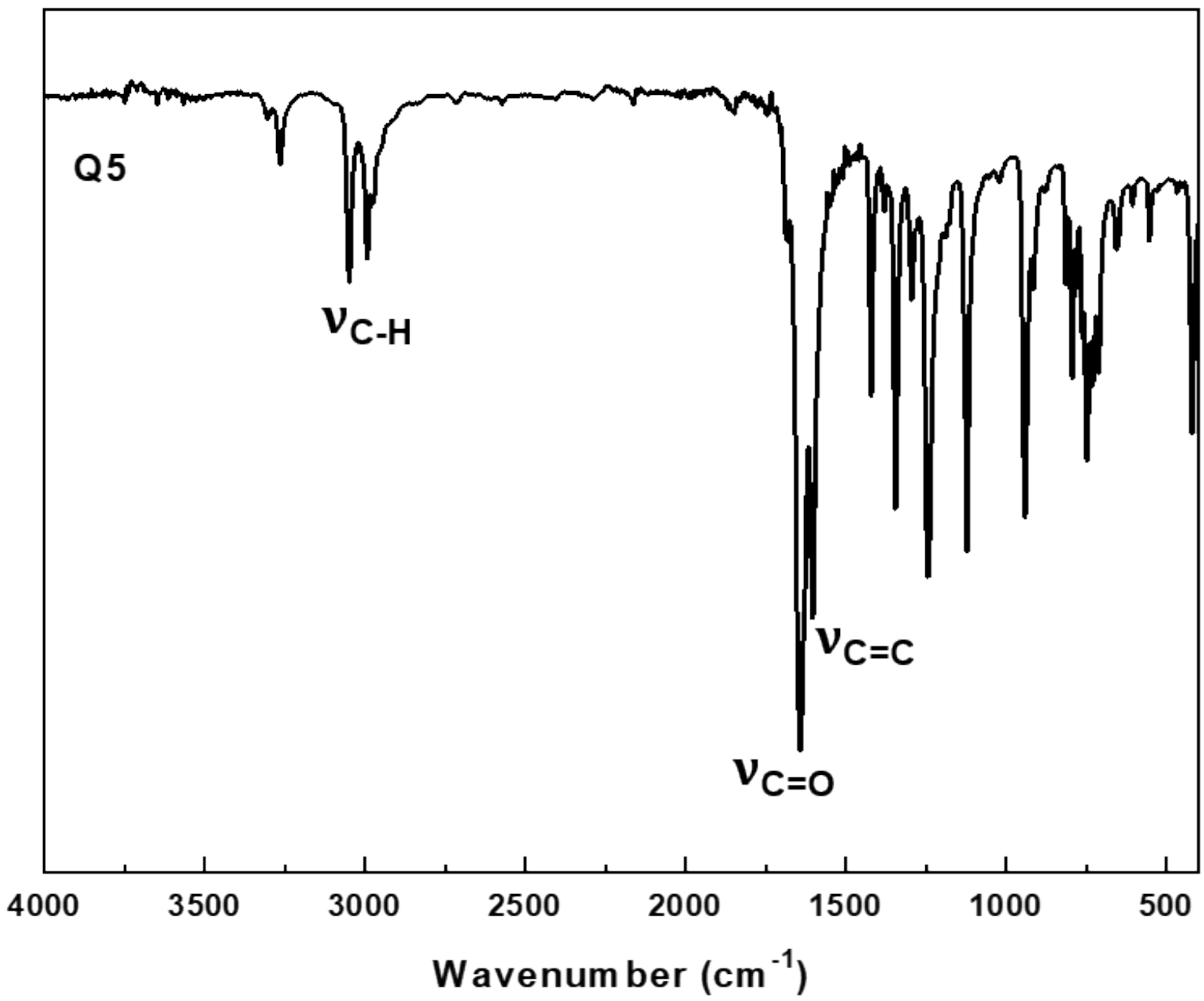

Figure S3. FT-IR spectra of Q5.

Section IV. High-performance liquid chromatography separation.

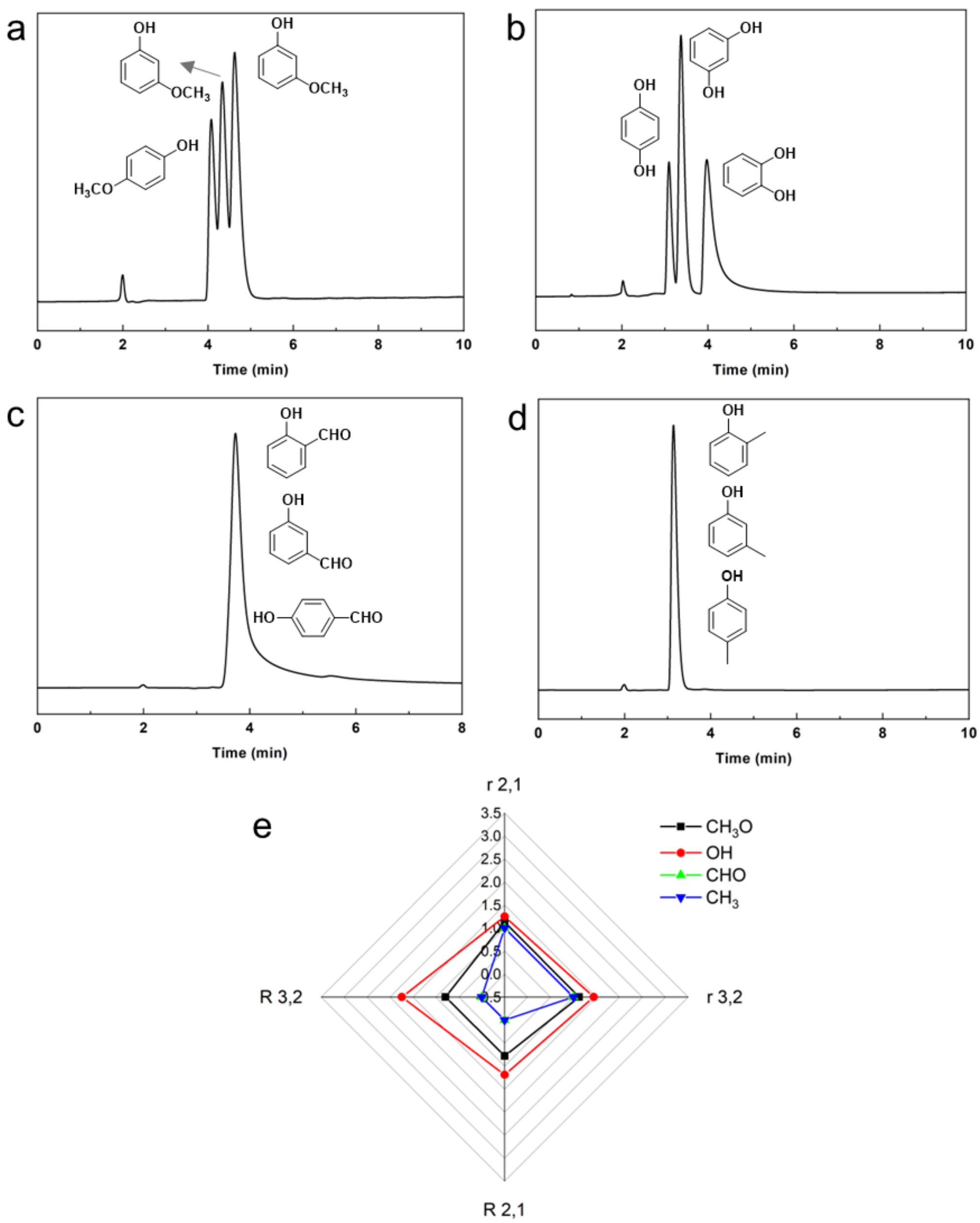

Figure S4. (a, b, c, d) Separation of hydroxyl position isomers on Q5-Sil packed column. (e) Effect of hydroxyl positional isomers on Q5-Sil packed column.

Table S2. Retention factors, selective factors and resolution of hydroxyl positional isomers.

Analytes	Elution order	*k*	α	R
Methoxyphenol	p	1.04		
	m	1.17	1.13	0.78
	o	1.31	1.12	0.79
Dihydroxybenzene	p	0.548		
	m	0.687	1.25	1.19
	o	0.987	1.44	1.74
Hydroxybenzaldehyde	p	0.865		
	m	0.865	1.00	0
	o	0.865	1.00	0
Hydroxytoluene	p	0.568		
	m	0.568	1.00	0
	o	0.568	1.00	0

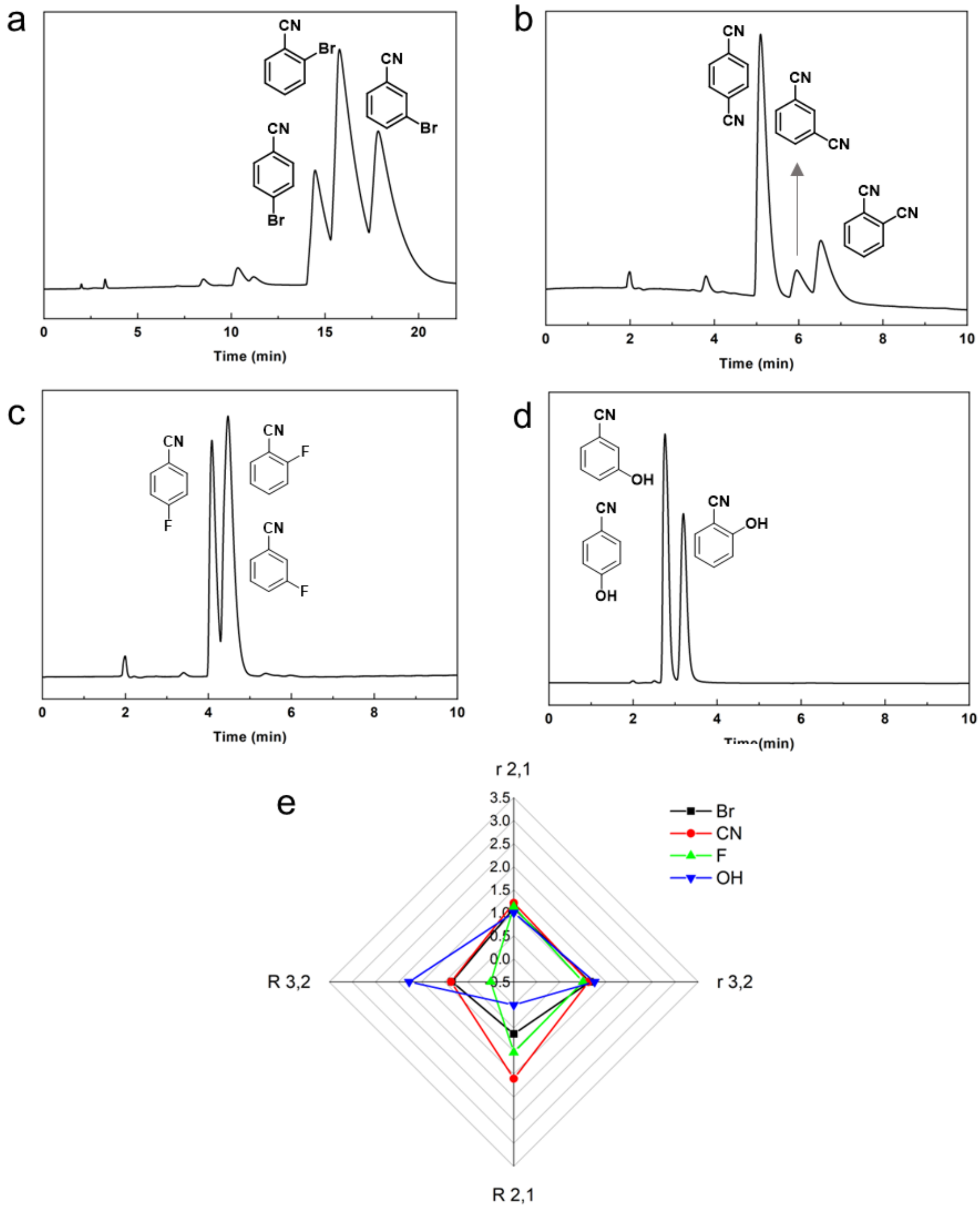

Figure S5. (a, b, c, d) Separation of nitrile positional isomers on Q5-Sil packed column. Mobile phase: (a) MeOH/H_2O (5:5); (b) MeOH/H_2O (6:4); (c) MeOH/H_2O (6:4); (d) MeOH/H_2O (7:3). (e) Effect of nitrile positional isomers on Q5-Sil packed column.

Table S3 Retention factors, selective factors and resolution of nitrile positional isomers.

Analytes	Elution order	*k*	*α*	R
Bromobenzonitrile	p	6.23		
	o	6.89	1.10	0.63
	m	7.92	1.15	0.84
Dicyanobenzene	p	4.10		
	m	4.95	1.21	1.60
	o	5.52	1.12	0.86
Fluorobenzonitrile	p	3.08		
	m	3.47	1.13	1.03
	o	3.47	1.00	0
Hydroxybenzonitrile	p	1.76		
	m	1.76	1.00	0
	o	2.20	1.25	1.77

Table S4 Retention factors, selective factors and resolution of nitro positional isomers.

Analytes	Elution order	*k*	*α*	R
Dinitrobenzene	o	0.87		
	p	1.38	1.58	2.94
	m	1.76	1.27	1.62
Nitrobenzonitrile	p	0.88		
	m	1.10	1.25	1.25
	o	1.37	1.25	1.33
Nitrophenol	m	0.57		
	p	0.76	1.35	1.88

	o	0.90	1.19	1.20
Nitrobenzaldehyde	o	0.18		
	p	0.25	1.38	0.20
	m	0.32	1.28	0.20

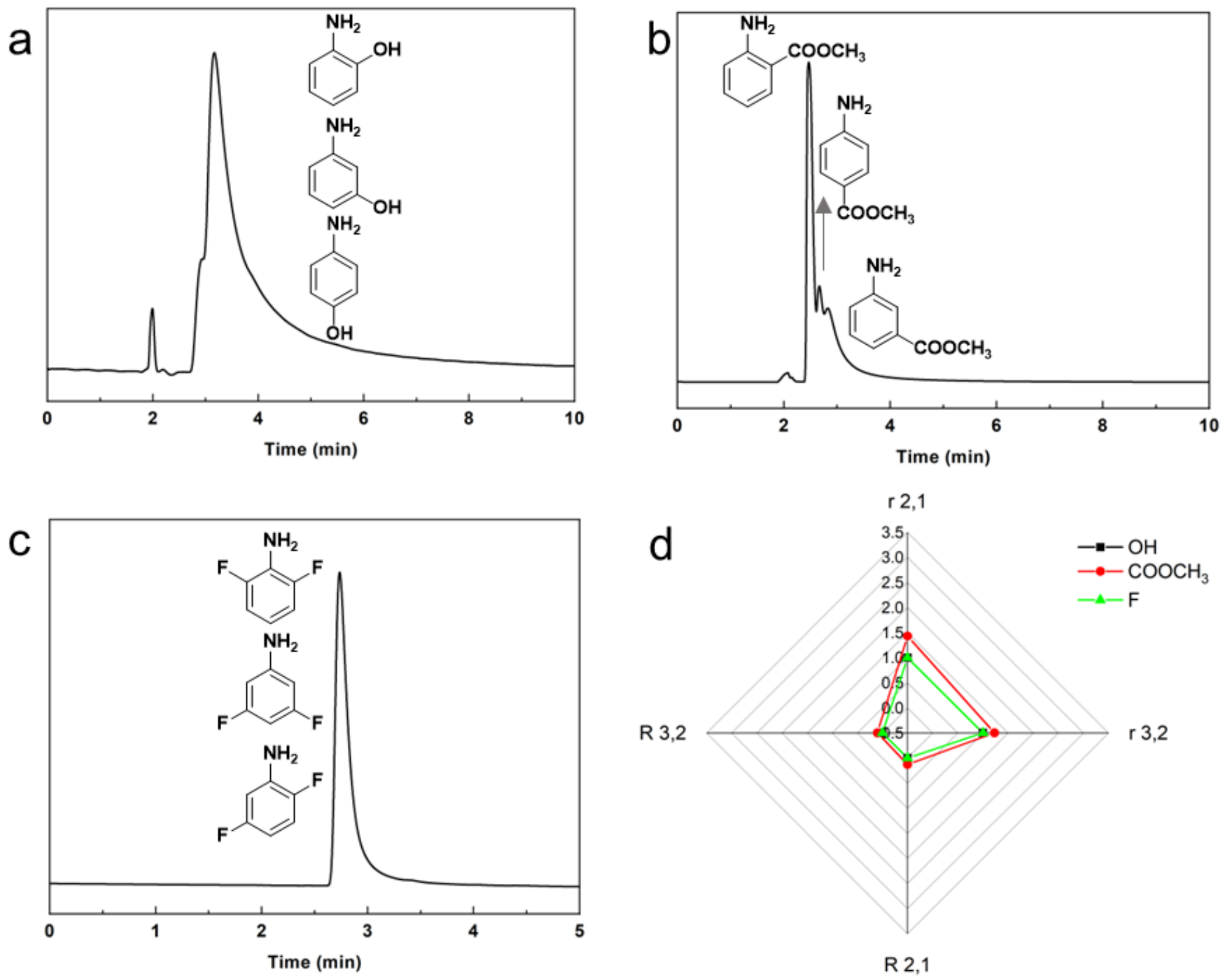

Figure S6. (a, b, c, d) Separation of amino positional isomers on Q5-Sil packed column. Mobile phase: (a) MeOH/H_2O (5:5); (b) MeOH/H_2O (6:4); (c) MeOH/H_2O (6:4); (d) MeOH/H_2O (7:3). (e) Effect of amino positional isomers on Q5-Sil packed column.

Table S5 Retention factors, selective factors and resolution of amino positional isomers.

Analytes	Elution order	*k*	α	R
Aminophenol	p	0.582		

	m	0.582	1.00	0
	o	0.582	1.00	0
Aminobenzoate	p	0.234		
	m	0.334	1.43	0.13
	o	0.410	1.23	0.10
Difluoroaniline	p	0.368		
	m	0.368	1.00	0
	o	0.368	1.00	0

Table S6. Retention factors, selective factors and resolution of eight aromatic nitro-explosives.

Analytes	*t*	*k*	α	R
nitrobenzene	9.3	2.735	—	—
2-nitrotoluene	12.5	4.020	1.47	2.21
4-nitrotoluene	15.5	5.225	1.30	2.21
3-nitrotoluene	16.6	5.667	1.08	0.63
m-dinitrobezene	20.0	7.032	1.24	1.34
2,4,6-trinitrophenylmethylnitramine	23.1	8.277	1.18	0.97
2-amino-4,6-dinitrotoluene	28.4	10.406	1.26	1.61
trinitrotoluene	31.3	11.570	1.11	0.87

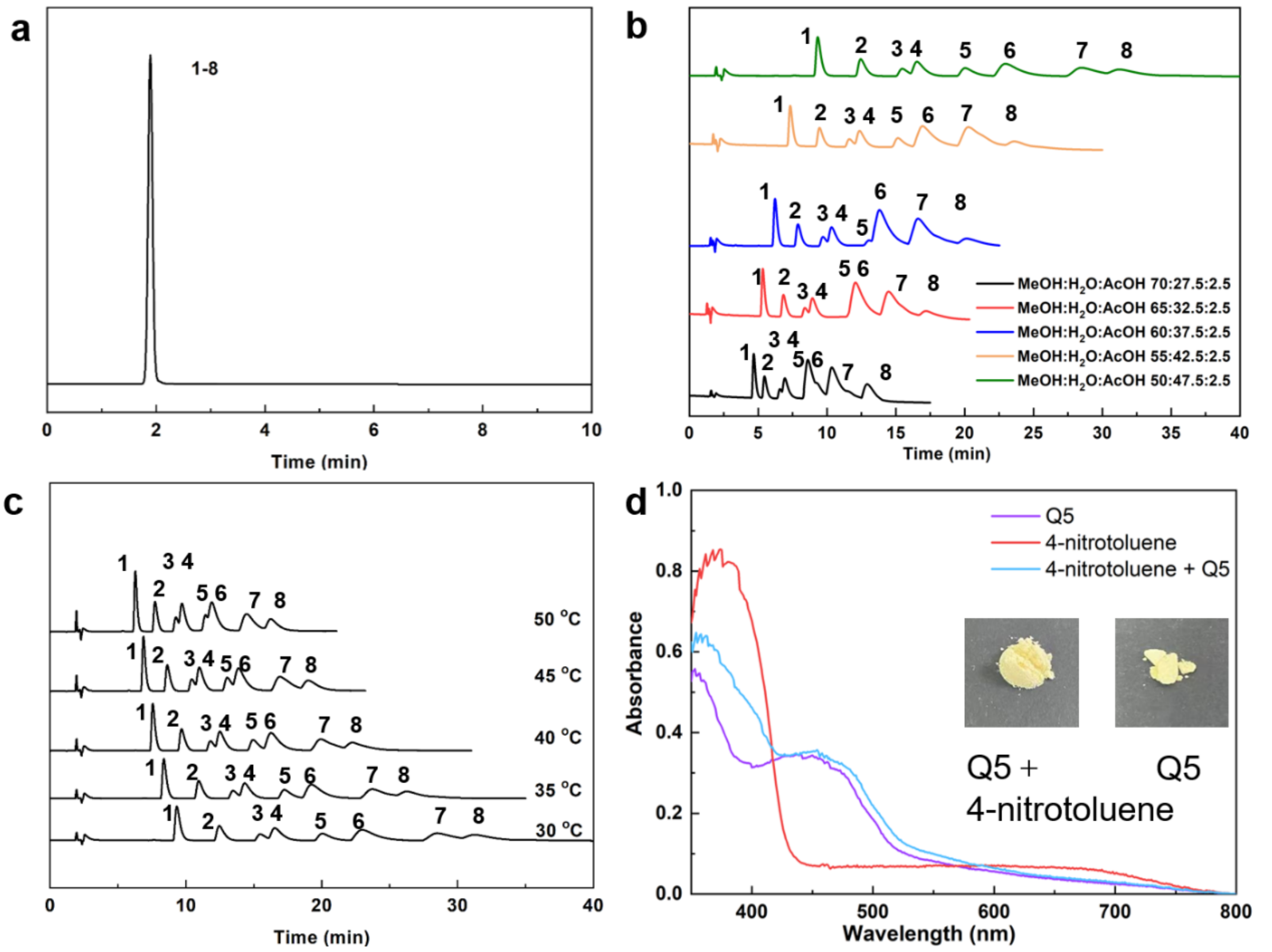

Figure S7. (a) Chromatogram of NH_2-Sil packed column (4.6 mm×150 mm, 5 μm) for LC separation of explosives under MeOH:H_2O:HAc (50%:47.5%:2.5%) at a velocity of 1.0 mL/min. (b) Effect of the mobile-phase composition on the separation of eight aromatic nitro compounds on the Q5-Sil-packed column. The MeOH/H₂O/AcOH ratios were 70:27.5:2.5, 65:32.5:2.5, 60:37.5:2.5, 55:42.5:2.5, and 50:47.5:2.5 (v/v/v). (Peak 1: nitrobenzene, Peak 2: 2-nitrotoluene, Peak 3: 4-nitrotoluene, Peak 4: 3-nitrotoluene, Peak 5: m-dinitrobezene, Peak 6: 2,4,6-trinitrophenylmethylnitramine, Peak 7: 2-amino-4,6-dinitrotoluene, Peak 8: trinitrotoluene) (c) Effect of temperature on LC separations of explosives on Q5-Sil packed column. (Peak 1: nitrobenzene, Peak 2: 2-nitrotoluene, Peak 3: 4-nitrotoluene, Peak 4: 3-nitrotoluene, Peak 5: m-dinitrobezene, Peak 6: 2,4,6-trinitrophenylmethylnitramine, Peak 7: 2-amino-4,6-dinitrotoluene, Peak 8: trinitrotoluene) (d) Q5, Q5 and 4-nitroluene grind solid under natural light photos. UV-Vis absorption

spectrum of Q5, 4-nitrotoluene, mixture of Q5 and 4-nitrobenzene.

Table S7. Thermodynamic parameters for LC separation of explosives on Q5-Sil packed column.

Analytes	ΔH (kJ mol^{-1})	ΔS (J mol^{-1} K^{-1})	ΔG (kJ mol^{-1})	R^2
nitrobenzene	-21.8 ± 0.8	-39.1 ± 1.8	-10.0 ± 0.2	0.988
2-nitrotoluene	-24.3 ± 0.9	-44.0 ± 1.9	-10.9 ± 0.3	0.992
4-nitrotoluene	-24.9 ± 1.0	-44.0 ± 2.0	-11.6 ± 0.6	0.993
3-nitrotoluene	-25.6 ± 1.2	-68.8 ± 1.7	-25.5 ± 0.5	0.993
m-dinitrobezene	-26.1 ± 1.1	-68.6 ± 1.9	-26.0 ± 0.4	0.994
2,4,6-trinitrophenylmethylnitramine	-30.2 ± 1.3	-80.9 ± 2.6	-30.0 ± 0.7	0.996
2-amino-4,6-dinitrotoluene	-30.4 ± 1.5	-79.6 ± 2.2	-30.2 ± 0.6	0.996
trinitrotoluene	-30.5 ± 1.7	-79.0 ± 2.1	-30.3 ± 0.5	0.997

References:

1. T. Ogoshi, S. Kanai, S. Fujinami, T.-a. Yamagishi and Y. Nakamoto, *J. Am. Chem. Soc.*, 2008, **130**, 5022-5023.
2. T. Ogoshi, T. Aoki, K. Kitajima, S. Fujinami, T.-a. Yamagishi and Y. Nakamoto, *J. Org. Chem.*, 2011, **76**, 328-331.
